# Osteology

**Published:** 1898-02

**Authors:** 


### Osteology.

How many bones in the human face?
Fourteen when they're all in place.
How many bones in the human head?
Eight, my boy, as I've often said.
How many bones in the human ear?
Three in each, and they help to hear.
How many bones in the human spine?
Twenty-six like a climbing vine.
How many bones in the human chest?
Twenty-four ribs, and two the rest.
How many bones in the shoulder bind?
Two in each—one before, and one behind.
How many bones in the human arm?
In each one, two in each forearm.
How many bones in the human wrist?
Eight in each if none are missed.
How many bones in the human hand?
Five in each with many a band.
How many bones in the fingers ten?
Twenty-eight, and by the joints they bend.
How many bones in the human knees?
One in each, the kneecap please.
How many bones in the human hip?
One in each like a dish they dip.
How many bones in the human thigh?
One in each and deep they lie.
How many bones from the leg to the knee?
Two in each and plain to see.
How many bones in the ankle strong?
Seven in each but none are long.
How many bones in the ball of the foot?
Five in each, as the palms were put.
How many bones in the toes half-a-score?
Twenty-eight, and then no more.
And now altogether these many bones fix?

And they count in the body two hundred and six.
And then we have the human mouth
Of upper and under thirty-two teeth.
And now and then a bone I should think
That forms on a joint, or to fill up a chink.
A sesamoid bone, or a wormian we call
And now we may rest for we've told them all.
—*Indian Medical Record.*

BUTLER, PA., is one of those hamlets given ephemeral fame by some unique circumstance. The oddity that pushes Butler into prominence for a moment is the bequest of Col. McKee, who devised that $2,000 should be placed in trust for the Butler School Board, the interest to be used in keeping clean and giving the proper care to the teeth of the public-school pupils whose parents or guardians are unable to bear the expense. A proviso of the bequest is that cleanliness of teeth is to be taught in the hygienic course of study, and the School Board has accepted the condition.

